# Effect of using nano-particles of magnesium oxide and titanium dioxide to enhance physical and mechanical properties of hip joint bone cement

**DOI:** 10.1038/s41598-024-53084-2

**Published:** 2024-02-03

**Authors:** Safaa Gamal, Mina Mikhail, Nancy Salem, Mohamed Tarek El-Wakad, Reda Abdelbaset

**Affiliations:** 1 Biomedical Engineering Department, Faculty of Engineering, Helwan University, Cairo, Egypt; 2 Mechatronics Engineering Department, Canadian International College, Cairo, Egypt; 3 Biomedical Engineering Department, Faculty of Engineering and Technology, Future University, Cairo, Egypt

**Keywords:** Biomedical engineering, Biomaterials

## Abstract

In this work, the effect of adding Magnesium Oxide (MgO) and Titanium Dioxide (TiO_2_) nanoparticles to enhance the properties of the bone cement used for hip prosthesis fixation. Related to previous work on enhanced bone cement properties utilizing MgO and TiO_2_, samples of composite bone cement were made using three different ratios (0.5%:1%, 1.5%:1.5%, and 1%:0.5%) w/w of MgO and TiO_2_ to determine the optimal enhancement ratio. Hardness, compression, and bending tests were calculated to check the mechanical properties of pure and composite bone cement. The surface structure was studied using Fourier transform infrared spectroscopy (FTIR) and Field emission scanning electron microscopy (FE-SEM). Setting temperature, porosity, and degradation were calculated for each specimen ratio to check values matched with the standard range of bone cement. The results demonstrate a slight decrease in porosity up to 2.2% and degradation up to 0.17% with NP-containing composites, as well as acceptable variations in FTIR and setting temperature. The compression strength increased by 2.8% and hardness strength increased by 1.89% on adding 0.5%w/w of MgO and 1.5%w/w TiO2 NPs. Bending strength increases by 0.35% on adding 1.5% w/w of MgO and 0.5% w/w TiO_2_ NPs, however, SEM scan shows remarkable improvement for surface structure.

## Introduction

Total hip arthroplasty (THA) is an artificial joint replacement operation with highly recorded treatment success rates^1^. The percentage of people undergoing this operation may reach 34% for over 65 age with around 498 thousand recorded cases worldwide in 5 years^2^. Bone cement is usually used for artificial joint fixation in THA operations, especially with people suffering from bone diseases such as osteoporosis^3^. Statistics reveal that more than 50% of THA operations failure is related to a deficiency in the bone cement properties^4,5^. Around 42.3% of bone cement failure may occur because of aseptic loosening and leakage. Around 9.6% due to instability. Around 90% of loosening, leakage, and instability of bone cement is due to limited bone cement mechanical properties^6^. Compression and bending strengths can clarify the bone cement mechanical strength^7^ that protects the bone from breaking or collapsing as a result of human activity force^8^. The previous research illustrated the effect of adding Titanium Dioxide (TiO_2_) and Magnesium Oxide (MgO) in improving bone cement mechanical properties^9,10^. The advantages of adding TiO_2_ particles to bone cement include higher elasticity, lower cytotoxicity, good cytocompatibility with osteoblasts, improved radiopacity, and improved mechanical qualities (compression and bending strengths)^11,12^. Also, adding MgO particles directly improves osteoblast adhesion^13,14^, antibacterial effect, cytocompatibility with osteoblasts, and potential for joint repair and fixation^15^. MgO was also found to enhance the bone cement bending modulus and hence the mechanical properties^16,17^. The impact of adding MgO in bone treatment is highlighted by surface deterioration, which produces a 30% increase in moving force due to bone osseointegration^18^.

Additives in nanoscale are trendy enhancement materials due to their small-scale effects, insensitivity to temperature, and good tribological characteristics. They offer superior properties and enhanced bone cell functions when compared to their micron-sized equivalents^6^. Combining one or more types of nanoparticles such as metal oxide, with one or more polymer composites results in a new class of nano-composites^19^. Particularly, the incorporation of polymers with nanoparticles of magnesium oxide (MgO NPs) and nanoparticles of titanium dioxide (TiO_2_ NPs) exhibits excellent physical and mechanical properties^20^. For TiO_2_ NPs and MgO NPs, the previous work clarified that TiO_2_ NPs with a ratio of 1% enhance bone cement mechanical properties significantly^21,22^, and MgO_2_ NPs with a ratio of 1% enhance nodules of calcium producing a high rate of osteogenic gene levels^23,24^.

Keeping the fundamental chemical structure of bone cement has reduced the ratio of NPs additions as previously described. To reduce chemical reactions that may result in a new composite with unneeded qualities, the number of additional ingredients is normally limited to one or two at most. In other words, targeted mechanical strength with selected precise additive ratios may have a detrimental impact on other mechanical parameters^25,26^.

This study aims to enhance the compression and bending strengths to reduce loosening and leakage problems of bone cement in THA operations, by using TiO_2_ and MgO materials in nanoparticles. In order to preserve the primary chemical structure of the bone cement, several ratios of both additive materials are examined that do not exceed 2% w/w. The field emission scanning electron microscopy (FE-SEM) and Fourier transform infrared spectroscopy (FTIR) are utilized to study the morphology of pure bone cement compared to composites with different ratios. Physical properties of all bone cement samples are illustrated through temperature, porosity, and degradation calculation, and mechanical properties through compression, hardness, and bending testing.

## Methodology

**Figure 1:**
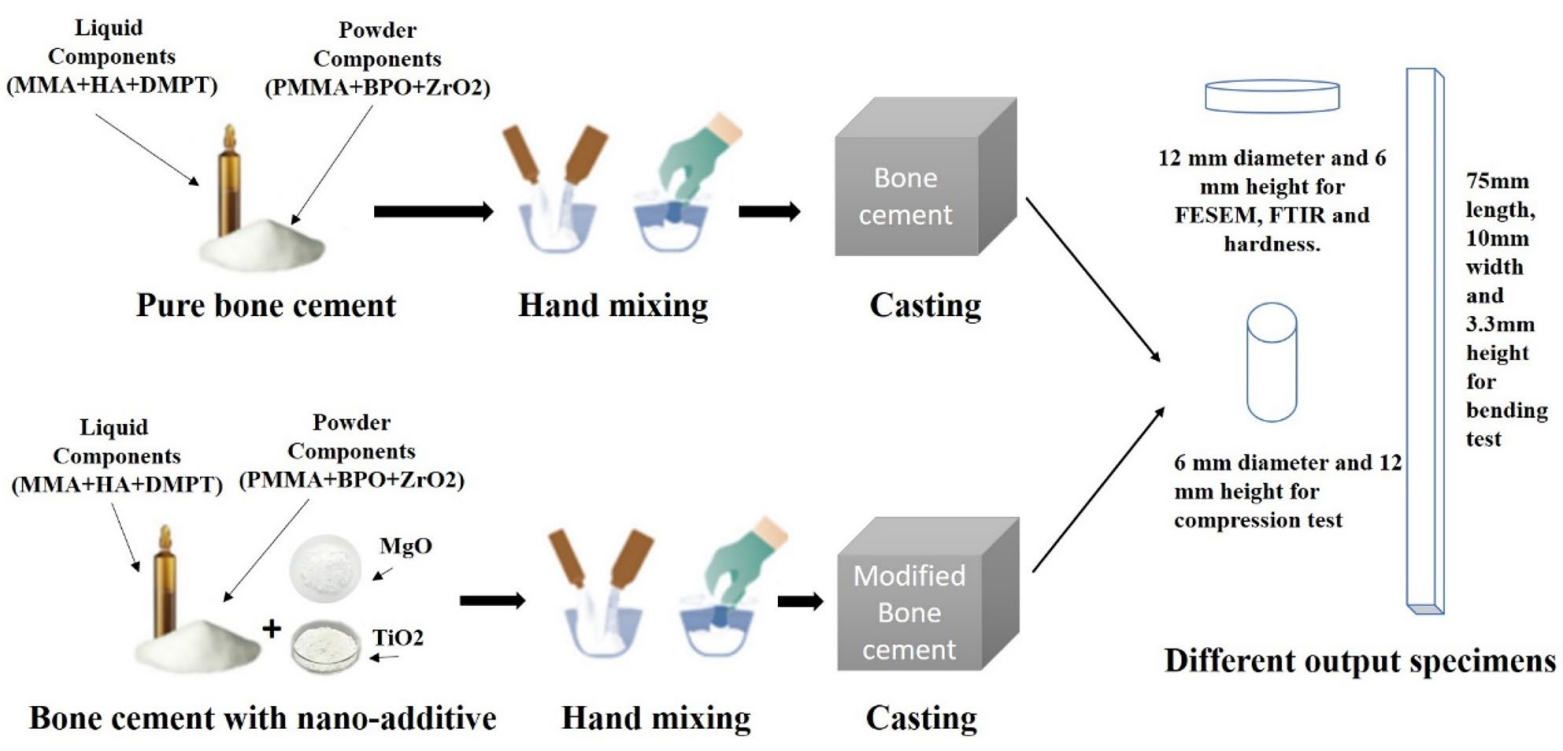
Steps to prepare the pure and composite bone cement.

Several stages are involved in the manufacture of bone cement composites as shown in Fig. 1. First, prepare the pure bone cement components either in powder or liquid form. Second, prepare the additive materials in nano size. Finally, prepare the bone cement composite with the required ratios of additive materials.

### Preparation of bone cement

Bone cement consists of powder (Polymer) and liquid (monomer) which is capable of polymerizing at room temperature^27^.

The Powder components of bone cement usually consist of Copolymers, initiators, and radiopaque materials. The copolymers are based on the substance polymethyl methacrylate (PMMA)^28^. The initiator is benzoyl peroxide (BPO)^29^ and the radiopaque is zirconia dioxide (ZrO_2_) particles^9^.

The liquid components of bone cement consist of monomer, accelerator, and stabilizer materials. The monomer component is methyl methacrylate (MMA) which is used to polymerize the PMMA^30^. The accelerator used is N, N-Dimethyl para-toluidine (DMPT), it is used to allow polymerization at room temperature, it has a special physical property that allows work at − 30 °C temperature which makes the polymerization to start at room temperature, it also adds to the adhesive property that matches with bone cement application^31,32^. The stabilizer inhibitor hydroquinone is used to prevent premature polymerization from exposure to light or high temperature during storage^33–35^.

The viscosity of bone cement varies between low, medium, and high viscosity. It depends on runny time, setting time, and bone cement components ratio. In this study, the high-viscosity bone cement which has a doughy, no runny, and ready state is used in THA operations^36,37^.

The preparation of bone cement starts with mixing the powder materials and liquid materials related to the ratios shown in Table 1. Then adding the powder and liquid materials together and mixing by hand in time not exceeding 30 s mixing time^38^. Powder and liquid components are mixed at an approximate ratio of 2.02: 1 to start a chemical reaction called polymerization, which forms bone cement^39^.

**Table 1:** Component of different commercial bone cement.

Powder/g (40.85)	
Poly methyl methacrylate %w/w	86.9
Benzoyl Peroxide %w/w	0.86
Zirconia dioxide (%w/w)	12.24
Liquid /ml (20.00)	
Methyl Methacrylate (%w/w)	99.35
N–N Dimethyl-p-Toluidine (%w/w)	0.65
Hydroquinone (ppm)	50

#### PMMA preparation

**Figure 2:**
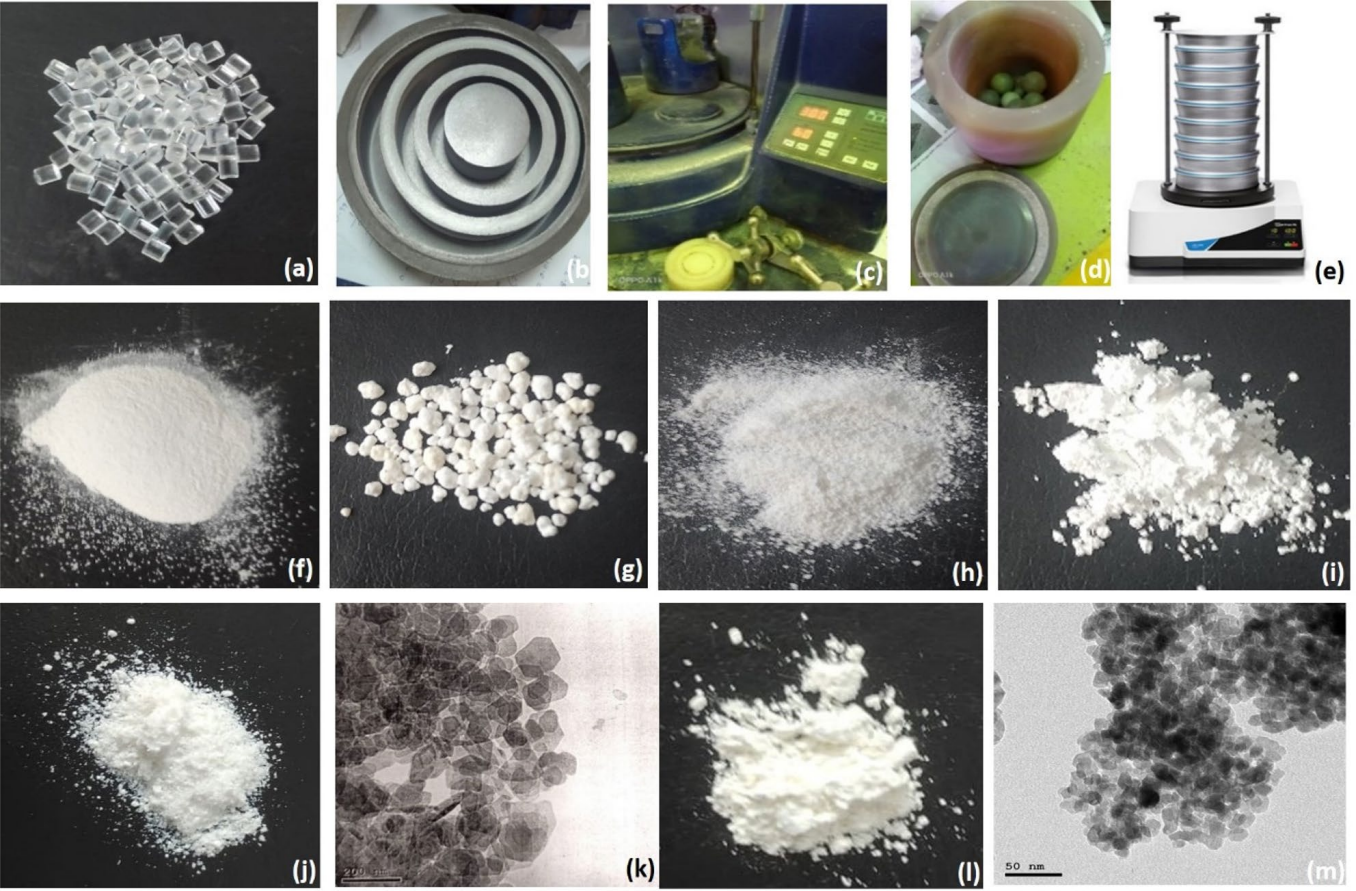
Preparations of bone cement and additive powder materials (**a**) The granule form of PMMA, (**b**) The grading, (**c**)The ball mill device, (**d**) The cup of ball mill device with ammonia balls, (**e**) Vibratory sieve shaker, (**f**) the < 50-micron size of PMMA, (**g**) BPO 1–2 mm grain, (**h**) BPO after grindery and sieve in vibratory sieve shaker, (**i**) ZrO2 powder, (**j**) The powder of MgO NPs (**k**) TEM micrographs of MgO NPs in 200 nm magnification, (**l**) The powder of TiO_2_ NPs and (**m**) TEM micrographs of TiO_2_ NPs in 100 nm magnifications.

The powder components copolymers of bone cement are based on the substance PMMA, which is found commercially in granule form as shown in Fig. 2a, but it must be in < 50-micron size to allow mixing and polymerization of bone cement^28^. The preparation of PMMA starts by grinding PMMA as shown in Fig. 2b to reach a granule size of 1 mm, then using the ball mill device with an ammonia ball for 7–8 h, with a 20-min break each 1 working hour till the PMMA granule size reaches < 50 microns, the rotating speed ball mill device was 300 rpm as shown in Fig. 2c, d. To check the size of PMMA and all powder materials the vibratory sieve shaker is used and it is shown in Fig. 2e. Figure 2f shows the powder of PMMA micron size.

#### BPO and ZrO_2_ preparation

BPO is collected in 1–2 mm diameter size, a specific steel spoon is used to grind the BPO grains till reaching 50 or less micro size, and the vibratory sieve shaker is used to check for the size of BPO, Fig. 2g shows the commercial size of BPO and Fig. 2h shows the > 50 micro size grains, the BPO allows starting of polymerization with low free radicals that keep low cytotoxicity to osteoblasts^29^. The radiopaque used is ZrO_2_ particles, commonly found in 2-micron size as shown in Fig. 2i, it has a direct effect in radiopaque that allows tracking and assessment of bone cement after fixing artificial joints, by focusing the view with X-ray scan^9,9,9^.

### Preparation of additive material

Previous research nominated that the total amount of additive percentage not exceed 5% of the total powder amount, for keeping the main chemical structure of bone cement.

Related to previous work that is mentioned in the introduction section “Methodology”, the ratio of 1% of Mg NPs and TiO_2_ NPs recorded enhanced properties of bone cement when used separately, the recommended ratio used in the proposed bone cement composite varies around 1% w/w (between 0.5%, 1%, and 1.5%) in the different samples as clarified in Table 2^40,41^. That is not to exceed 2% w/w in total of both additives to keep the main structure of bone cement.

**Table 2:** Samples components with different ratios.

Sample	PMMA (%)	MgO NPs (%)	TiO_2_ NPs (%)
S_1	100		–
S_2	98	0.5	1.5
S_3	98	1	1
S_4	98	1.5	0.5

Sample 1 (S_1) is prepared as pure bone cement by mixing the powder and liquid ratios as clarified in Table 1. Sample 2 (S_2) includes 2% (0.5% MgO NPs and 1.5 TiO_2_ NPs) of additive ratio, after mixing 100 g of powder components (PMMA, BPO, and ZrO2) related to Table 1, then replacing 2 g by the additive component and mixing well, finally adding liquid ratio for the 100 g of powder to prepare high viscosity bone cement composite. Sample 3(S_3) includes 2% (1% MgO NPs and 1 TiO_2_ NPs) and sample 4 (S_4) includes 2% (1.5% MgO NPs and 0.5 TiO_2_ NPs) powder ratio.

#### Preparation of nano magnesium oxide

MgO NPs are prepared using a top-down approach that starts by breaking down the magnesium large pieces till getting the required nano size as shown in Fig. 2j. The targeted average size of 15 ± 2 nm is checked by transmission electron microscope (TEM) which is performed on (JEOL JEM-2100) high-resolution TEM at an accelerating voltage of 200 kV as shown in Fig. 2k.

#### Preparation of nano titanium dioxide

TiO_2_ NPs are also prepared in 15 ± 2 nm average size with Quasi-Spherical like shape and Anatase crystal structure as shown in Fig. 2l, the top-down approach is used to get target size, then they are checked by a transmission electron microscope (TEM) which is performed on (JEOL JEM-2100) high-resolution TEM at an accelerating voltage of 200 kV as shown in Fig. 2m.

### Morphology structure method

In this study, Fourier transform infrared spectroscopy and field emission scanning electron microscopy are used for surface check.

#### Fourier transform infrared spectroscopy (FTIR)

Fourier transform infrared (FTIR) indicates the group function of each sample. A small piece of each composite sample is used to apply the FTIR test which is performed using Bruker ALPHA II device with IR Affinity-1 at room temperature, ranges from 500 to 4000 cm^−1^ mid-IR source and KBr beam splitter are used in the test. The X-axis shows wave number (cm^−1^ spectrum), and the Y-axis shows absorbance units^42^.

#### Field emission scanning electron microscopy (FE-SEM)

**Figure 3:**
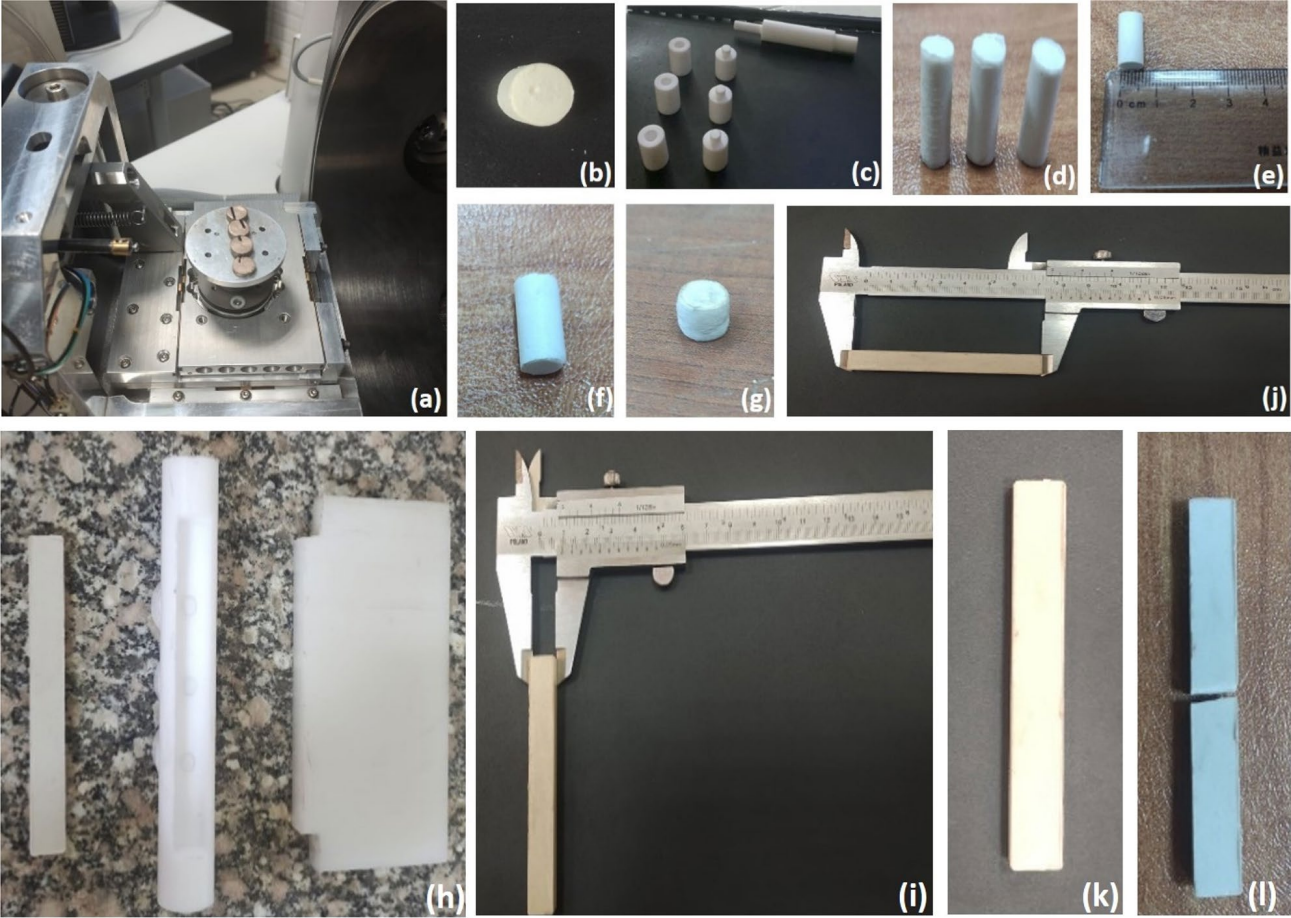
(**a**) The covered specimens in SEM chamber, (**b**) cement specimen with hardness test notch, (**c**)The Teflon molds as three parts: base, body and presser, (**d**, **e**) Compression strength specimen with 6 mm diameter and 12 mm height, (**f**) bone cement compression specimen before and (**g**) specimen after compression test, (**h**)The Teflon mold parts: base and body part of bending test, (**i**) Bending specimen width 10 mm, (**j**) Bending specimen length 75 mm, (**k**) Bone cement bending specimen before bending test and (**l**) specimen after the bending test.

The microstructure and surface morphology of bone cement specimens are examined using a scanning electron microscope (FE-SEM) at an accelerated voltage of 30 kV, chamber pressure 582 Pa, gum pressure 3.65 e^−7^ Pa, and emission current 120 µA. The 12 mm diameter with 5 mm thickness sample was coated with a thin layer of gold for each composite, to allow heat build-up in samples and improve electrostatic charging during the scanning, Fig. 3a shows the samples after gold coating in SEM device^42,43^.

### Physical tests

For physical changes in pure and composite bone cements, porosity and degradation were calculated and compared, also temperature change was measured through the polymerization process.

#### Porosity and degradation degree (DR) tests

The porosity (P) of bone cement specimens is calculated due to Eq. (1). Three specimens for each of the four samples (S_1, S_2, S_3, and S_4) were used with 6 mm diameter and 10–12 mm height dimensions after 2 days of preparation. After calculating the initial specimen weight W_0_ as the average of the three specimens, the specimens were immersed in simulation body fluid SBF for 28 days at room temperature 23 °C, in day 28 the swelling specimen weight W_1_ was measured, then the dry specimen weight W_2_ was measured after complete drying^44,45^.

1 $$p \left(\mathrm{\%}\right)=\frac{\left({W}_{2}-{W}_{0}\right)}{{W}_{2}-{W}_{1}}100\mathrm{\% }$$

Degradation (D) of bone cement specimens is calculated due to Eq. (2), 48 specimens (12 for each of S_1, S_2, S_3, and S_4) were used with 6 mm diameter and 9–12 mm height dimensions 2 days after preparation. First specimen weight W_0_ was measured, and then specimens were immersed in SBF for 28 days in an incubator with 37° temperature degree, in each of the 7, 14, 21, and 28 days three specimens from each sample ratio were extracted and the average dry weight W_2_ of the three samples was calculated after complete drying^44,46^.

2 $$D \left(\%\right)=\frac{\left({W}_{0}-{W}_{2}\right)}{{W}_{0}}100\%$$

The porosity and degradation tests were done using an incubator (Binder, model ED-S 56 made in Germany) and 4 Digits Balance (Sartorius laboratory Entries 224-1S) to measure weight.

#### Setting temperature calculation

A thermocouple of E type was used to measure the bone cement temperature. Three specimens with 68 mm diameter and 10 mm thickness for each of S_1, S_2, S_3, and S_4 were prepared according to ISO5833 standard^47^. The probe of the thermocouple was installed on the specimen and the temperature was measured synchronously with time. The setting temperature is calculated according to Eq. (3):

3 $${T}_{set}=\frac{{T}_{max}+{T}_{amb}}{2}$$

where T_set_ is the setting temperature, T_max_ is the maximum temperature and T_amb_ is the ambient temperature^23^.

### Mechanical test

For studying pure and composite samples morphology and different mechanical properties, several tests were applied including.

#### Compression test

The Computerized Control machine is used for specimen compression test, related to ISO 5833:2002, a Teflon mold shown in Fig. 3c was prepared to produce compression test specimens with dimensions 6 mm for diameter and 12 mm for height as clarified in Fig. 3d,e^21,48^. 12 specimens were tested considering 3 specimens for each composite. The compressive strength is calculated according to Eq. (4) ^49,50^:

4 $$compressive\, strength {\sigma }_{f}=\frac{F}{A}$$

where F is fracture load (N) and A is the initial cross-sectional area (mm^2^).

Figure 3f clarifies the compression test specimen before the test and Fig. 3g after compression.

#### Hardness test

Resistance of deformation is one of the important properties that must be tested in bone cement to keep the THA and HA operations successful, hardness test shows the degree of material deformation resistance^51^. 12 mm diameter and 6 mm height specimen is prepared for each composite to be put under a diamond probe in a Vickers micro-hardness device. Figure 3b clarifies the specimen after the hardness test. To calculate the material hardness value, Eq. (5) is used:

5 $$Hardness\, vickers HV=1.354 \times \frac{F}{{\left(\frac{d1+d2}{2}\right)}^{2}}$$

#### Three-point bending test

According to ISO 5833:2002, a Teflon mold as shown in Fig. 3h is prepared to produce rectangular bending test specimens with dimensions 75 mm, 10 mm, and 3.3 mm as clarified in Fig. 3i,j^11^. Specimens were tested in AUTOMAX MULTITEST Computerized Control machine.

The bending strength of three-point bending is calculated due to Eq. (6):

6 $$Bending\, stregth \sigma = \frac{3{P}_{f}L}{b{d}^{2}}$$

The fracture stress can be calculated due to Eq. (7) ^17^.

7 $$Fracture\, stress {\sigma }_{fs}= \frac{3{P}_{f}S}{2b{d}^{2}}$$

where p_f_ is the fracture load (N), L is the distance of the inner points of load (mm), S is the standard loading span for the three-point bend specimen, b is the width of sample (mm) and d is the height of the sample (mm)^21^.

Figure 3k clarifies the bending test specimen before the test and Fig. 3l the specimen after bending.

## Results

In this section, the effect of adding MgO and TiO_2_ Nps to pure bone cement using the specified percentages in Table 2 is illustrated according to the experimentation methodologies shown in section “Methodology”. First, change in bone cement morphology structure is studied using Fourier transform infrared spectroscopy and field emission scanning electron microscopy. Second, changes in physical properties of setting temperature, porosity, and degradation are studied. Last, the effect on the mechanical properties of hardness, compression, and bending is shown.

### Surface structure results

**Figure 4:**
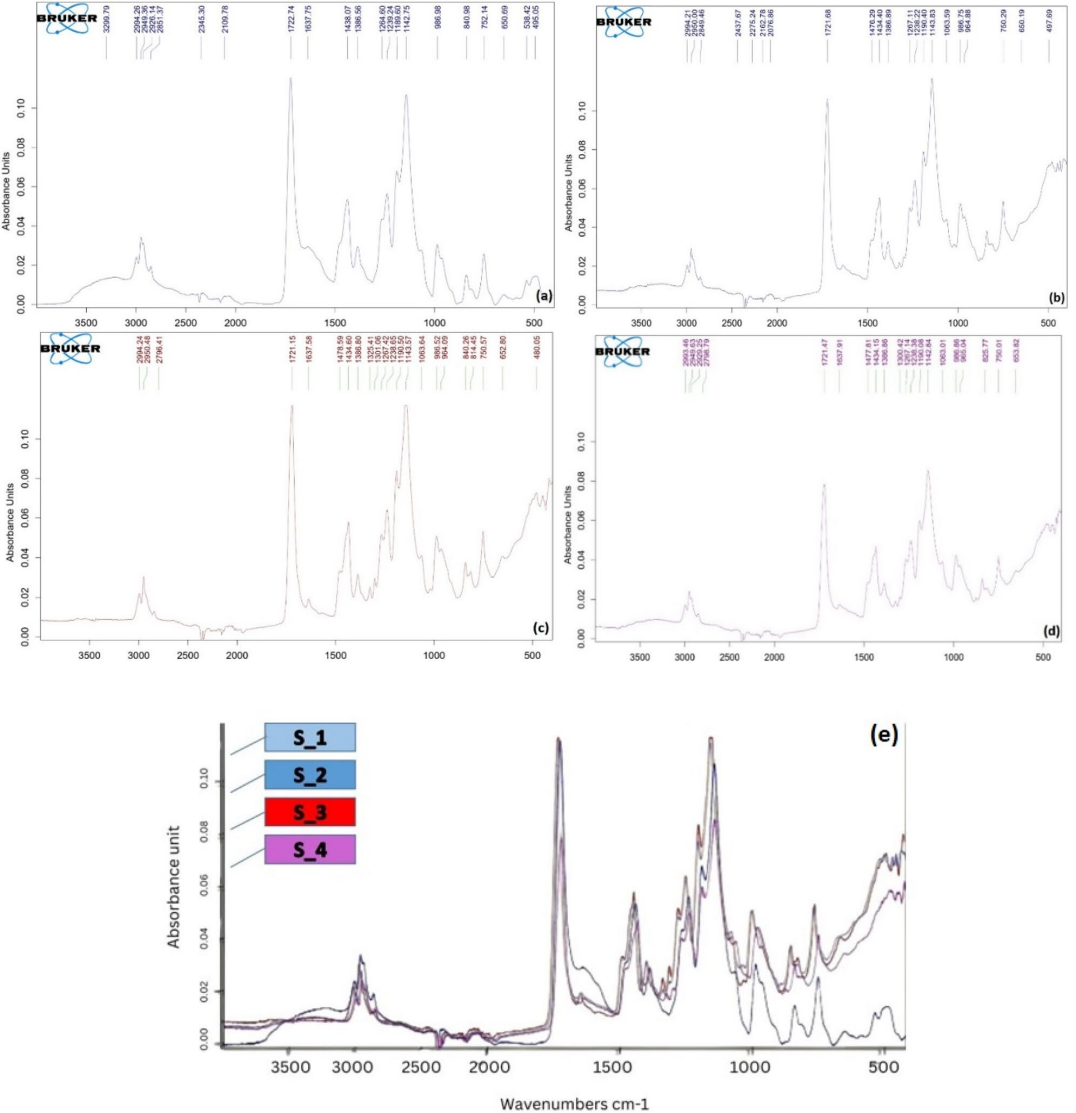
The FTIR of (**a**) S_1, (**b**) S_2, (**c**) S_3, (**d**) S_4 in 2 days after preparation, and (**e**) The FTIR of a combination of four samples in one graph.

The FTIR spectra absorption of S_1 is clarified in Fig. 4a, sample absorption peaks of IR spectra range from 4000 to 500 cm^−1^ are recorded at many ranges such as 2949, 1722, 1438, 1142, 986, and 495 wavelengths that correspond to the main characteristic ranges of bone cement^52^.

Figure 4b–d clarifies the FTIR spectra absorption of S_2, S_3, and S_4 with additional peaks appearing in curves due to the composite bone cement with different ratios of MgO NPs and TiO_2_ NPs, which is clarified in Fig. 4e showing a combination of graphs of pure and composite bone cement samples in same graph^53,54^.

**Figure 5:**
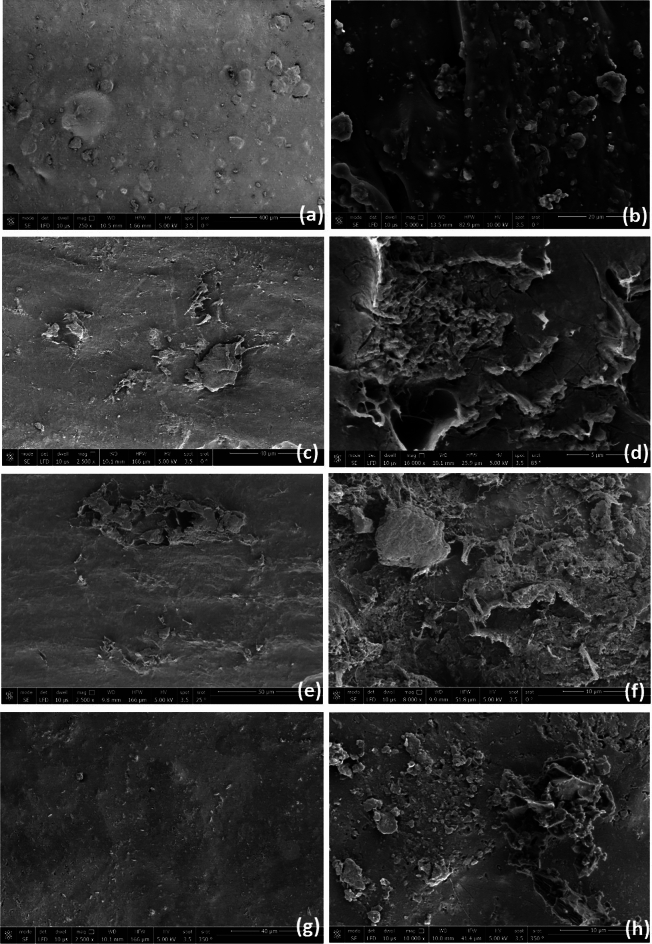
The FE-SEM in magnification 2500× of (**a**) S_1, (**c**) S_2, (**e**) S_3 and (**g**) S_4. The FE-SEM inmagnification 10,000× of (**b**) S_1, (**d**) S_2, (**f**) S_3 and (**h**) S_4.

After immersing the bone cement in the hip to fix, its surface has direct contact with human bones, surface morphology gives an impression of future attaching reaction, and FE-SEM scan produces clear surface properties accurately. Figure 5 clarifies the surface morphology of pure and composite bone cement with nano additives in two magnification scans of 2500× and 10,000×. Figure 5a for S_1 shows a rough pure bone cement surface, however, the roughness decreases slightly in Fig. 5c,e,g for composite samples S_2, S_3, and S_4 at 2500×*g* scan, which results from including the nano additive materials that fill in the micron cavities between the bone cement grains. However, at 10000×, the FE-SEM image clarifies in Fig. 5b the pure bone cement that includes small granules of PMMA mixed with other components of bone cement. In Fig. 5d,f,g FE-SEM image clarifies that bio-composite microstructures were homogeneous which will have an effect in enhancing the mechanical properties. Additionally, the FE-SEM with a high magnification image of the composite includes pores in the surface structure that allow bone intercellular to link when the bone cement is immersed in the body^55^.

## Physical and mechanical test results

The polymerization of bone cement starts at an ambient temperature of 22 °C, it takes 30 s to mix then the thermocouple is immersed in the specimens and the maximum temperature is calculated at different time intervals during polymerization. The maximum temperature is calculated during 590 s after polymerization as the maximum value of temperature^56^.

**Figure 6:**
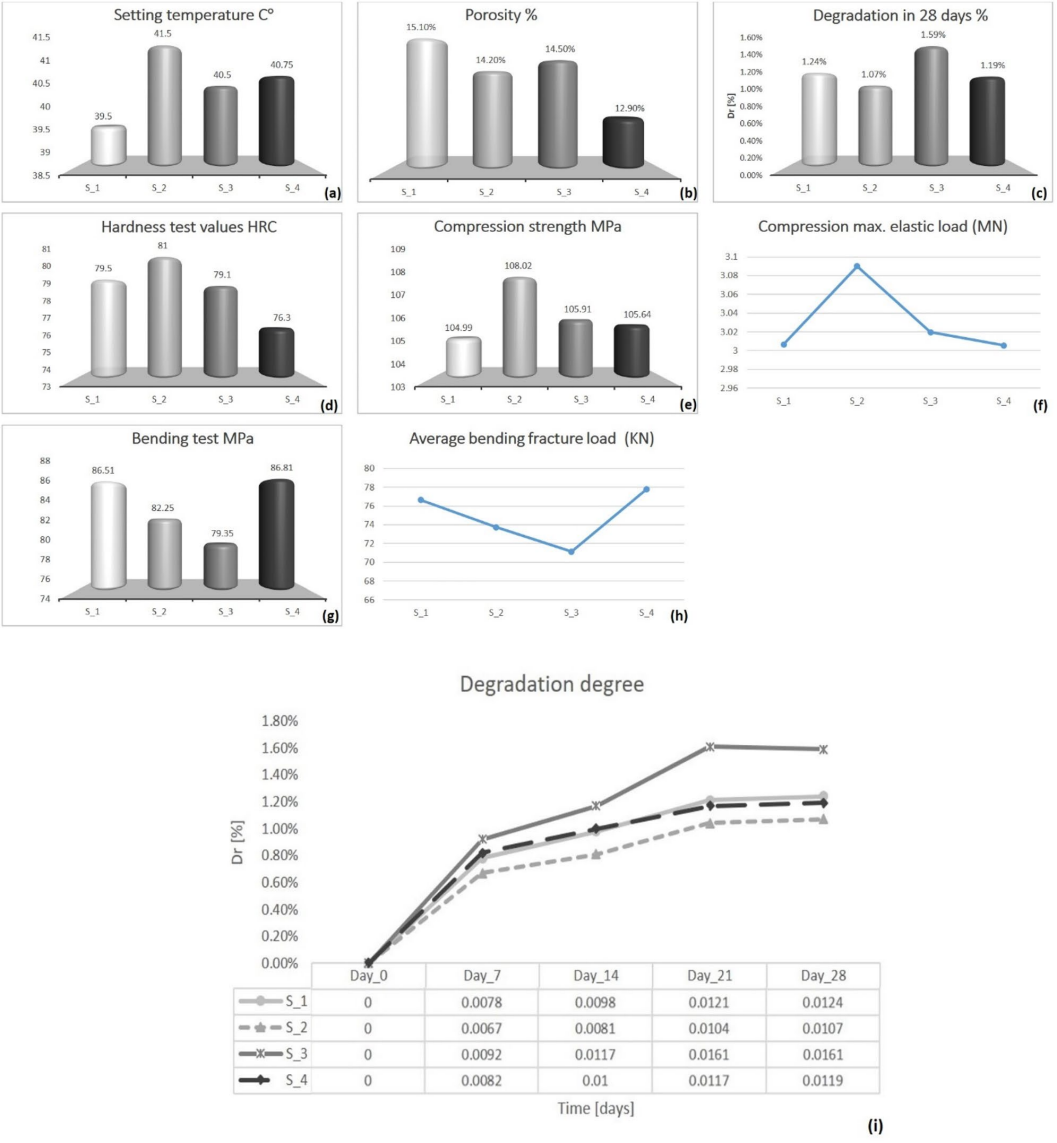
(**a**) The setting temperature of S_1, S_2, S_3, and S_4, (**b**) porosity percentage of the four tested samples, (**c**) degradation rate after 28 days for the four samples, (**d**) the Vickers micro-hardness test values for the four samples, (**e**) compression test values for the four samples, (**f**) compression maximum load for the four samples, (**g**) bending strength in MPa for the four samples, (**h**) Average bending fracture load for the four samples and (**i**) the degradation of the four samples after 0, 7, 14, 21 and 28 days.

The setting temperature of bone cement specimens is shown in Fig. 6a, S_2 recorded a maximum temperature of 41.5 °C, followed by S_4, S_3, and S_1 at 40.75 °C, 40.5 °C, and 39.5 °C, respectively. The variation in setting temperature is small due to the low ratio of additive components which keeps the main setting temperature.

Porosity is an important property of bone cement as it represents the pores where bone cells will probably form and grow however, however relatively high porosity ratios may pose a danger affecting other mechanical properties. The porosity acceptable varying range for bone cement is between 5 and 15%^57^. Samples S_1, S_2, S_3, and S_4 recorded 15.1%, 14.2, 14.5 and 12.9% of porosity, respectively. This indicates that additive nano-particles in S_2, S_3, and S_4 fill in some cavity space between micro-particles producing low porosity in them as shown in Fig. 6b.

Degradation has hidden meaning in bone cement fixation as it is described as a non-biodegradable material, where lower degradation levels indicate a relatively higher living time of the bone cement implant in the body. Results in Fig. 6c clarify that the degradation rate increases with the increase of MgO NPs and the least degradation is recorded in S_2 with increasing TiO_2_ NPs, this indicates that S_2 will keep a longer living time. Degradation percentage after 28 days in solution represents an important value for material behavior, S_1, S_2, S_3, and S_4 recorded 1.24%, 1.07%, 1.59%, and 1.19% respectively as shown in Fig. 6c,i.

The body motion mechanism in the hip joint produces friction which may scratch the bone cement, however, harness value indicates the resistance of the material to scratch. The Vickers micro-hardness test on the four samples recorded 79.5, 81, 79.1, and 76.3 HRC respectively as shown in Fig. 6d. This indicates that the increase of TiO_2_ NPs is directly proportional to the hardness value. However, increasing MgO NPs is considered inversely proportional to hardness.

Compression strength is one of the important mechanical properties of bone cement due to the mechanical stress that occurs on the surface of the bone cement during body stand or movement^8^. The sample compression strength for S_1, S_2, S_3, and S_4 recorded 104.99, 108.02, 105.91, and 105.64 MPa respectively with the highest value in S_2 according to ISO 5833:2002 as clarified in Fig. 6e. Figure 6f depicts the maximum compression load for the four samples, with a value that varies from 3 to 3.09 MN.

The bending test allows the calculation of bone cement fracture load and bending strength, three specimens for each of the four samples shown in Table 2 were tested for bending, and the average bending strength was calculated for each sample. The four samples, S_1, S_2, s_3, and S_4, recorded average bending strength 86.51, 82.25, 79.35, and 86.81 MPa bending strength respectively as clarified in Fig. 6g. Figure 6h descripes the fracture load that is noticed at bending test for the four samples , with a value that varies from 71 to 77.81 KN.

The specimens also recorded average fracture strength of 85.04, 70.9, 73.25, and 87.41 MPa respectively.

## Discussion

As clarified from the results, using additive Nps to pure bone cement reduces previously faced problems of loosening, leakage, and limited bone cement properties in artificial hip joint fixation^6^. TiO_2_ Nps are used due to their good mechanical strength that enhances composite mechanical properties. MgO Nps are used because of their role in human bone formation which enhances attachment between bone cement and human bone^58^. Both Materials TiO_2_ and MgO are used in nanoparticle form to benefit from the nano properties of spreading and distributing within the mixture, especially since the additive amount in bone cement composite will not exceed 2% w\w for both materials. Ratios of composite additives are kept as small as possible to maintain the main chemical structure of bone cement. The surface and morphology structure of pure and composite bone cements are studied through characterization with FT-SEM and FTIR, which clarify that no new phase was found in composite bone cement, that may back the usage of small and nano forms of additive materials.

The viscosity of composite bone cements in S_2, S_3, and S_4 had no change of high viscosity type, which appears through the continued presence of the doughy bone cement during preparation, and keeping the setting time range ± 2 s over pure bone cement setting time.

The setting temperature, degradation ratio, and porosity are important properties that identify the composite behavior. For setting temperature the change in value was very little while keeping the same protocol of using bone cement in operation. Degradation ratio results match those of pure bone cement with a slight change in the range of less than 0.01%. The porosity of composite bone cement falls in the acceptable range that varies between 5 and 15% w\w. Porosity has a hidden important task as it allows for conductivity to cell growth and bone formation, this directly will lead to enhancing mechanical properties of bone cement after being impressed in the human body^45^.

To check for enhanced mechanical properties, the Compressive strength in human cortical bone varying between 90 and 230 MPa is checked, in the other side, the commercial bone cement mechanical compression strength usually equals 100 ± 5 MPa^59^. Raising bone cement compression strength is a major scope of this study. All of the experimented composite bone cement samples succeeded in enhancing bone cement compression strength, especially specimens with additive 0.5%w/w of MgO and 1.5%w/w TiO_2_ NPs enhanced compression strength by 3.03 MPa.

For bone cement bending strength that is produced from the bending test, ranges from 45 to 90.5 MPa in pure bone cement^60^. The composite strength of bone cement in this study matches the normal range with values between 79 and 86 MPa. For Flexural stress properties that are produced from the 3-point bending test, results also match suitable values varying between 70 and 87 MPa^17^. The Maximum load for compression and the fracture load for bending test were recorded in Table 3.

**Table 3:** Summarization of the average bending fracture load and compression maximum load.

Sample	Bending fracture load (KN)	Average bending fracture load	Compression maximum load (M)	Average compression maximum load
S_1	76.5	76.65	2.994	3.0067
	75.45		3.039	
	78		2.987	
S_2	74	73.72	3.106	3.090
	73.55		3.085	
	73.6		3.079	
S_3	70	71.12	3.039	3.0197
	71.35		2.998	
	72		3.022	
S_4	78	77.81	3002	3005.3
	75.77		2998	
	79.65		3016	

Bone cement with additive MgO and TiO_2_ NPs shows general improvement in endurance with a maximum compression load, it also recorded suitable bending fracture load as clarified in Table 3. Three specimen values for each of the four experiments are tested for tested for bending fracture load and maximum compression load and average values are calculated as clarified in Table 3.

Based on previous experimentation and results that are summarized in Table 4, it is recommended to apply vitro experiments to S_2 with 0.5%w/w of MgO and 1.5%w/w TiO_2_ additive ratio, with proven desirable compression strength, porosity, degradation ratio, and surface structure, as well as acceptable bending strength.

**Table 4:** Summarization of the results of mechanical and physical properties of cortical, pure, and composite bone cement.

Measured properties	Standard ^11,25^ ^26,61^	Bone cement range	Samples with different ratio			
			S_1	S_2	S_3	S_4
Compression strength (MPa)	90–230	100 ± 5	104.99	108.02	105.91	105.64
Hardness strength (HRC)	–	–	79.5	81	79.1	76.3
Bending strength (MPa)	180	45–90.5	86.51	82.25	79.35	86.81
Setting temperature (°C)	37 °C	37–120 °C	39.5	41.5	40.5	40.75
Maximum temperature (°C)			56	52.5	54	49
Degradation %	–	–	1.24%	1.07%	1.59%	1.19%
Porosity %	Average 3.5%	5–15%	15.1%	14.2%	14.5%	12.9%

## Conclusion

The objective of this article is to examine how adding MgO and TiO_2_ NPs to bone cement can improve both its mechanical and physical qualities. To demonstrate the impact of adding additives on the final composite qualities, three composite samples were created by mixing various proportions of the two addition materials with pure cement. Samples were inspected for morphology as well as mechanical and physical characteristics. The experimental results confirm the following considerations:

- The composite’s FT-SEM morphology is more homogeneous than pure bone cement due to the addition of nano-sized components, resulting in surface pours that facilitate adhesion between human bone and cement.
- The FTIR curve of pure bone cement shows strong peaks at critical locations, indicating that the primary chemical bonds remained constant. However, due to the modest ratio of nano additions, the FTIR of composite materials includes supplementary peaks that demonstrate the presence of MgO and TiO_2_ NPs additives while maintaining the primary bone cement chemical wavelength peak unchanged.
- Composite bone cement has a setting temperature rise of no more than 2 °C higher than pure bone cement, which is considered acceptable.
- Adding TiO_2_ NPs reduces porosity by 0.5–2%, but MgO NPs causing a 2% drop. However, lowering porosity improves durability.
- The degradation percentage decreases by 0.17% with high TiO_2_ NPs, 0.05% with high MgO NPs, and increases by 0.35% with equal MgO and TiO_2_ NPs. The decrease in degradation immediately improves the mechanical characteristics of bone cement specimens.
- Adding MgO NPs (S_3 with 1.5% MgO NPs) reduces bone cement hardness by 3.2 HRC in, whereas adding TiO_2_ NPs (S_2 with 1.5% TiO_2_ NPs) enhances hardness by 1.5 HRC.
- The addition of 0.5%w/w MgO and 1.5%w/w TiO_2_ NPs to composite bone cement specimens increased compression strength by 3.03 MPa. Improved compression strength and hardness allow patients to withstand heavier loads without bone cement leakage.
- Adding 1.5% w/w of MgO and 0.5% w/w TiO_2_ NPs increased bending strength by 0.03 MPa. Other composite bone cement ratios resulted in a minor reduction of bending strength up to 4.5 MPa. However, bending strength is reduced by a reasonable amount, and the surface’s chemical structure is enhanced in comparison to pure bone cement structure, demonstrating a poured and homogenous surface that maintains the best adhesion between bone cement and human bone without loosening.

Consequently, to prevent bone cement loosening and leakage during total hip arthroplasty surgeries, it is advised to utilise a composite bone cement specimen containing 0.5%w/w MgO and 1.5%w/w TiO_2_ NPs.

Future research will examine fractures in bone cement layers caused by overloading, fatigue tests on pure and recommended specimens, and the recommended specimens in vitro experiments for recommended composite bone cement.

## Data Availability

The datasets used and analyzed during the current study are available from the corresponding author upon reasonable request.
