# Author Correction: Effect of using nano-particles of magnesium oxide and titanium dioxide to enhance physical and mechanical properties of hip joint bone cement

**DOI:** 10.1038/s41598-024-74392-7

**Published:** 2024-10-07

**Authors:** Safaa Gamal, Mina Mikhail, Nancy Salem, Mohamed Tarek El‑Wakad, Reda Abdelbaset

**Affiliations:** 1 Biomedical Engineering Department, Faculty of Engineering, Helwan University, Cairo, Egypt; 2 Mechatronics Engineering Department, Canadian International College, Cairo, Egypt; 3 Biomedical Engineering Department, Faculty of Engineering and Technology, Future University, Cairo, Egypt

Correction to: *Scientific Reports*10.1038/s41598-024-53084-2, published online 03 February 2024

In the original version of this Article, Mohamed Tarek El-Wakad was incorrectly affiliated with ‘Biomedical Engineering Department, Faculty of Engineering, Helwan University, Cairo, Egypt.’ The correct Affiliation is listed below.

Mohamed Tarek El-Wakad

Biomedical Engineering Department, Faculty of Engineering and Technology, Future University, Cairo, Egypt.

The original Article has been corrected.

